# Identifying contributing-factor configurations in reported bed falls: a Bayesian network–based exploratory study

**DOI:** 10.3389/fpubh.2026.1785239

**Published:** 2026-04-09

**Authors:** Mingxuan Wang, Yanhui Zhang, Jiuqun Li, Peitao Li, Tianyi Huo, Yuting Jiang, Quanying Qu, Baohua Li

**Affiliations:** 1 Department of Nursing, Peking University Third Hospital, Beijing, China; 2 Department of Nursing, Peking University Shenzhen Hospital, Shenzhen, China; 3 School of Nursing, Peking University, Beijing, China

**Keywords:** Bayesian network, bed fall, contributing factors, inpatient adverse events, patient safety

## Abstract

**Objective:**

This study aimed to explore the conditional dependency structure of contributing factors related to bed falls and to identify stable dependency configurations within reported bed falls incident reports using Bayesian network analysis.

**Methods:**

A retrospective analysis was conducted using 102 inpatient bed-fall incident reports collected from a tertiary hospital between 2014 and 2024. Twenty-two previously identified contributing factors were encoded as binary variables. Bayesian network structure learning was performed using a hill-climbing algorithm with the Bayesian Information Criterion, without imposing prior structural constraints. Structural stability was assessed through non-parametric bootstrap resampling. Conditional inference and posterior probability analyses were used to examine probability changes and co-occurrence patterns under specific clinical conditions.

**Results:**

Bootstrap analysis identified several structurally stable directed edges in the Bayesian network. Sudden changes in patient consciousness were repeatedly connected to medication-related hypotension and safety measure-related nodes, forming a central structural component of the network. A stable directed relationship was also observed between nighttime sedative use with toileting and unassisted bed exit. In addition, delayed patient assistance was more frequently linked to delayed responses by on-site caregivers than to complete caregiver absence. Together, these results indicate that reported bed falls are associated with a limited number of recurrent and structurally consistent contributing-factor configurations.

**Conclusion:**

Reported bed falls appear to arise from interactions among a small number of contributing factors under specific clinical contexts rather than from isolated causes. Bayesian network analysis offers a useful approach for identifying contributing-factor configurations and informing scenario-based fall prevention strategies in clinical nursing practice.

## 1 Introduction

Within hospital safety management systems, patient falls are among the most frequently reported adverse events (1). It is estimated that 20% to 60–70% of in-hospital falls are connected to “beds or bedside chairs,” with roughly half of these incidents occurring during patient transfers in and out of bed (2–4). Although most bed falls result in only minor injuries, nearly 5% may lead to severe consequences (5), posing an ongoing threat to patient safety. A scoping review by Ainani and Irwan systematically analyzed the factors associated with patient falls in hospitals and found that a considerable proportion of falls occur in the bedside environment. In one of the studies included in their review, approximately 42.1% of fall incidents occurred at the patient’s bedside, indicating that the bedside area is an important high-risk location for fall events (6). These findings highlight that the bedside environment represents a critical context for fall prevention and should receive greater attention in hospital safety management and nursing practice.

As a specific type of fall incident, bed falls exhibit unique mechanisms and require distinct prevention priorities. However, existing research and clinical practices often categorize them broadly under “falls,” failing to adequately address their independent contributing factors structure. Previous studies have primarily focused on identifying and categorizing general fall contributing factors at the hospital level, typically grouping them into environmental, patient, staff, and medical factors (6). While this approach provides valuable insights into the overall causes of hospital falls, it does not specifically examine the structural relationships among contributing factors in bed-related fall incidents. Commonly adopted preventive measures-such as bed rails, low-height beds, instructing patients not to get up unassisted, and hourly rounding-face practical challenges. Those patients most in need of assistance often struggle to comply due to clinical conditions, cognitive limitations, or urgent toileting needs (7).

Previous research on patient falls has mainly focused on identifying individual contributing factors or developing fall-risk assessment tools, while rarely examining the configurational patterns of multiple contributing factors within fall events, particularly in cases of bed falls. However, incident reports of bed falls typically contain multiple contributing factors occurring within the same event. For example, a fall may occur when a patient with impaired mobility attempts to get out of bed unassisted during nighttime while environmental conditions or nursing workload also play a role. In such situations, a bed fall is not attributable to a single factor but rather to the configuration of several factors within a specific clinical context. Understanding how these contributing factors co-occur in reported bed-fall events is therefore important for improving prevention strategies. Recent advances in the analysis of unstructured clinical text have enabled researchers to examine incident reports in greater detail. Using natural language processing techniques, Wang et al. (8) analyzed inpatient bed-fall incident reports and identified multiple categories of contributing factors, including patient-related factors, ward environment and equipment factors, medication-related factors, caregiver factors, and nursing practice factors. While this work provided a structured description of factors associated with bed falls, it did not explore how these factors are related to one another within reported events.

Bayesian networks provide a suitable framework for modeling such complex clinical risk systems. By representing contributing factors as nodes within a directed acyclic graph and learning conditional dependencies from observed data, Bayesian networks allow for the identification of key structural relationships and enable probabilistic inference under different clinical evidence patterns. This includes estimating how the probability of specific events changes when certain conditions are present, as well as identifying which related contextual conditions are most commonly associated when an adverse event has occurred (9–11). In healthcare research, Bayesian networks, as a class of probabilistic graphical models, have been widely used to represent conditional dependencies among multiple variables in complex systems. By modeling variables as nodes in a directed acyclic graph and estimating conditional probability relationships from observed data, Bayesian networks enable risk modeling and inference under conditions of uncertainty. In clinical and nursing research, this approach has been applied to the analysis of multivariable influence mechanisms and nursing-related adverse events, such as medication errors (9–13). Previous studies suggest that Bayesian networks can help uncover latent structural characteristics of complex risk systems and support probabilistic analysis of risk changes across different clinical scenarios. In recent years, Bayesian analytical methods have also been increasingly introduced into nursing research to address multivariable dependency and uncertainty (14).

Building on the previously established BERTopic-derived risk factor framework, the present study applies Bayesian network modeling to the same set of 22 bed-fall contributing factors, encoded as binary variables from 102 inpatient bed fall incident reports. Rather than rediscovering individual contributing factors, this study aims to reconstruct the structural dependency patterns among contributing factors observed in reported bed-fall incidents as a probabilistic system, addressing the following questions: (1) How contributing factors are conditionally related and organized within the network structure; (2) Which nodes occupy structurally central positions in the network; (3) How the probabilities of key events change under different observed clinical conditions.

Identifying contributing factors of bed falls helps nurses recognize high-risk patients earlier and supports hospitals in developing more targeted fall-prevention strategies. More importantly, compared with identifying single contributing factors, examining the dependency relationships among multiple factors and constructing risk configurations better reflects real clinical situations. In clinical practice, a single risk factor rarely leads directly to a bed fall. Instead, bed falls often occur when several contributing factors interact within a specific clinical context. Analyzing dependencies among multiple contributing factors can help identify high-risk situations that may repeatedly occur in clinical practice and improve the understanding of how different factors are related. This approach can also help identify key factors within the risk network. Because these factors may be conditionally associated with several other risks, they may serve as potential early warning signals and provide nurses with more proactive cues for risk recognition. In addition, understanding bed falls from the perspective of risk configurations may inform more scenario-based nursing interventions. Compared with general preventive measures targeting individual contributing factors, configuration-based analysis can support the design of more specific interventions for particular clinical situations, such as nighttime activity, medication-related conditions, or care-response processes. Such scenario-based strategies may improve the relevance and effectiveness of fall-prevention practices in bedside nursing care.

## 2 Method

The design and reporting of this study followed the Minimum Information for Medical AI Reporting (MINIMAR) checklist, which provides guidance to enhance transparency, reproducibility, and interpretability in clinical artificial intelligence research (15).

The present study aligns with the MINIMAR framework in several key aspects:

(1) The use of real-world clinical safety data derived from inpatient bed fall incident reports; (2) the application of an interpretable probabilistic modeling approach-Bayesian network analysis-to explore conditional dependencies among clinical contributing factors; (3) the explicit representation of model structure and assumptions through a directed acyclic graph, facilitating clinical interpretation; (4) the use of scenario-based and posterior probabilistic inference to support clinically relevant risk reasoning and decision support.

In accordance with the exploratory and hypothesis-generating nature of Bayesian network modeling, this study focused on structural learning and probabilistic inference rather than predictive performance evaluation. The following sections are therefore reported in line with the MINIMAR recommendations for transparency and interpretability.

### 2.1 Study design and data sources

This study is a retrospective, exploratory study aiming to further analyze the structural organizational characteristics and conditional dependencies of previously identified contributing factors for falls among hospitalized patients. The study data were derived from fall incident reports recorded in the adverse event reporting system of a tertiary general hospital. A total of 102 bed fall incidents among hospitalized patients between 2014 and 2024 were included in the study.

### 2.2 Definition and data encoding of contributing factors

#### 2.2.1 Source of the contributing-factor framework

The risk factor framework used in this study is derived from previous text mining research conducted on the same set of fall incident reports. This research systematically identified and summarized 22 contributing factors related to bed falls among hospitalized patients using natural language processing methods. These factors cover multiple dimensions, including patient status, ward environment and equipment, medication-related factors, caregiver factors, and nursing practice factors. This study does not generate or select new contributing factors, but rather conducts further structural modeling analysis based on this existing framework.

#### 2.2.2 Contributing-factor coding scheme

In order to facilitate quantitative modeling and result presentation, each of the 22 contributing factors associated with bed falls identified in the previous study was assigned a unique alphabetical code (A–V), forming a standardized variable coding framework for Bayesian network analysis.

The contributing factors included: A, improper use of bed rails; B, inadequate postoperative nursing education; C, older adult patients using the toilet alone; D, improper use of call bells; E, patient’s auditory and visual impairments; F, sudden change in patient’s consciousness; G, orthostatic hypotension induced by diuretic antihypertensive drugs; H, poor drug adherence; I, inadequate monitoring of sedated patients; J, caregivers failing to notice patient’s condition in time; K, combined use of sedatives and femoral nerve block analgesia pumps; L, unsuitable bed design; M, poor collaboration between healthcare providers; N, using sedative-hypnotic drugs and using the toilet at night; O, patient’s cognitive and communication impairments; P, lower limb weakness caused by femoral nerve block analgesia pumps; Q, irregular use of antiepileptic drugs; R, impaired consciousness not being effectively managed; S, caregiver absence leading to care deficiency; T, caregivers not assisting patients in time; U, getting out of bed without assistance after using sedative-hypnotic and analgesic drugs; and V, non-slip resistant slippers.

#### 2.2.3 Binary variable encoding process

In this study, the above 22 contributing factors were used as candidate variables for Bayesian network analysis and uniformly encoded as binary variables. The incident reports contained narrative descriptions of the bed-fall events, documenting the circumstances and contributing conditions surrounding each incident.

For each report, a risk factor was coded as “1” if it was explicitly documented in the report as contributing to the bed-fall event. A factor was coded as “0” indicates that the factor was not documented as contributing to the event in the original report. In the reporting system used in this study, factors were recorded when they were identified by the reporting nurse as contributing to the event. Therefore, factors not documented in the report were interpreted as not identified as contributing causes at the time of reporting. Coding was strictly based on the content recorded in the incident reports, and no additional clinical inference was introduced by the researchers.

To ensure coding reliability, two researchers independently performed the binary encoding based on the documented report content. Inter-rater agreement was assessed using Cohen’s kappa prior to consensus discussion (Cohen’s kappa = 0.787). Discrepancies were resolved through discussion, with a third researcher serving as arbitrator when necessary.

### 2.3 Bayesian network modeling and statistical analysis

#### 2.3.1 Bayesian network structure learning

This study uses a Bayesian network (BN) approach to model and analyze the conditional dependencies among contributing factors associated with bed falls. A Bayesian network is a probabilistic graphical model that represents conditional dependencies between variables using a directed acyclic graph (DAG) and characterizes uncertainty using conditional probability distributions (16).

The network structure learning employs the hill-climbing search algorithm, using the Bayesian Information Criterion (BIC) as the scoring function to balance model fit and structural complexity. This study did not introduce any theory-based prior structural constraints; the network structure was entirely learned from the data. To improve model stability and prevent overly complex network structures, the maximum number of parent nodes for each variable was restricted to three during the hill-climbing search process. The results are used for exploratory analysis and generating hypotheses about risk relationships, rather than for strict causal inference.

After the network structure was learned, parameter estimation was performed using Bayesian estimation implemented in the pgmpy library. Specifically, a Bayesian Dirichlet equivalent uniform (BDeu) prior with an equivalent sample size of 1.0 was applied to estimate conditional probability tables. This approach introduces Dirichlet smoothing, which helps mitigate potential zero-frequency problems that may arise in sparse binary datasets.

#### 2.3.2 Bootstrap-based structural stability analysis

Because Bayesian network structure learning can be sensitive to sampling variability, a bootstrap-based procedure was used to evaluate the robustness and reliability of the learned network structure. The bootstrap approach assesses whether the identified structural relationships remain consistent under repeated resampling of the data, thereby providing an estimate of the stability of the inferred edges.

Structure learning was repeated on multiple bootstrap resamples using the same hill-climbing algorithm with BIC scoring, and the occurrence frequency of each edge was calculated. Edges with higher bootstrap frequencies were considered stable structural components and were retained for subsequent inference analyses.

In this study, 200 bootstrap resampling iterations were performed to evaluate the robustness of the learned network structure. This number is consistent with common practice in bootstrap-based structural analysis (17). To assess convergence, exploratory analyses were conducted using larger numbers of bootstrap resamples. The frequencies of the most stable edges changed only minimally beyond 200 iterations, indicating that the stability estimates had converged at this level.

### 2.4 Network structure feature analysis

To identify key structural nodes in the fall risk system, a topological analysis of the learned Bayesian network was performed, calculating the in-degree and out-degree of each node. By analyzing the connection characteristics among nodes, it is possible to further understand the structural positions of different contributing factors within the overall risk network and the roles they may play.

### 2.5 Conditional inference and posterior analysis

#### 2.5.1 Conditional inference

Based on the constructed Bayesian network model, conditional probability inference is used to analyze the changes in the probability of risk events occurring under different clinical scenarios. Specifically, the value of a certain contributing factor is fixed, and the conditional probability changes of other related contributing factors in the network were calculated to simulate the probability distribution characteristics within the risk system under different conditions.

It should be noted that this analysis is based on conditional probability inference (conditioning) and is used for exploratory scenario analysis, not for strict causal “intervention.”

#### 2.5.2 Posterior probability analysis

To identify the contributing factors that are more likely to coexist when a specific adverse event or risk event occurs, a posterior probability analysis is further conducted. Given that a key node has been observed to occur, the posterior probability of related upstream contributing factors being “1” is calculated, thereby identifying the combination of contributing factors most likely to be activated in actual fall incidents.

All structural learning and inference analyses of Bayesian network models were performed in a Python environment, primarily using the pgmpy and other related probabilistic graphical model analysis libraries.

### 2.6 Ethical considerations

According to the ethical guidelines of the Institutional Review Board of Peking University Third Hospital, an exemption from informed consent was obtained (IRB00006761-M2024873).

## 3 Results

### 3.1 Bayesian network structure learning results

#### 3.1.1 Bayesian network structure learning

Based on 102 inpatient bed-fall incident reports, a total of 22 binary contributing-factor variables were included in the Bayesian network analysis. Network structure learning was performed using a hill-climbing algorithm with the Bayesian Information Criterion (BIC) as the scoring function, without imposing prior structural constraints.

**Figure 1:**
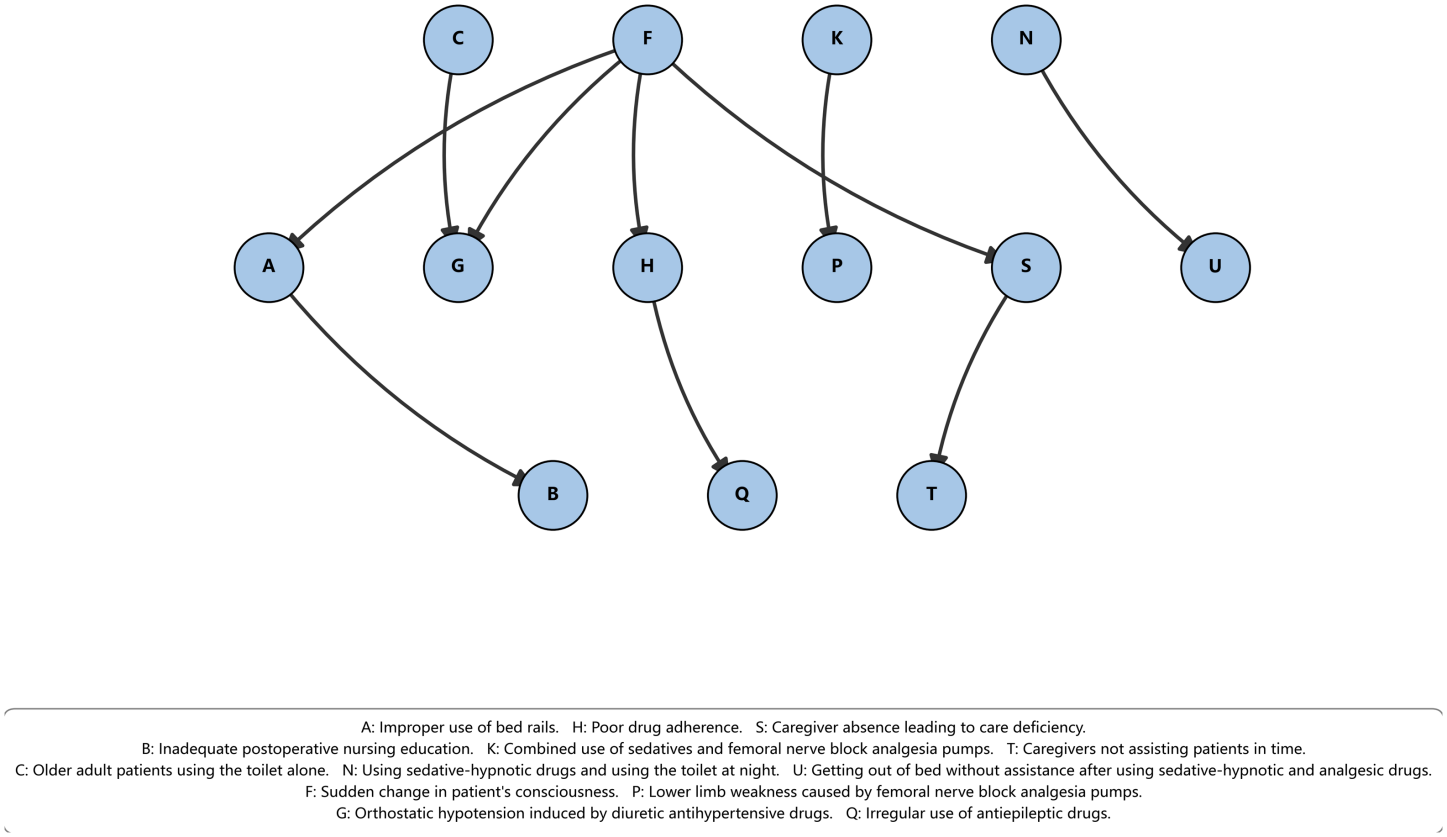
Bayesian network structure of bed fall-related contributing factors in hospitalized patients. This figure illustrates the learned Bayesian network describing conditional dependency relationships among contributing factors observed in reported bedside falls. The network highlights sudden change in patient consciousness (F) as a central node in the network, showing conditional associations with several related factors, including medication-related hypotension (G), improper use of bed rails (A), medication adherence issues (H), and caregiving-related deficiencies (S). A caregiving pathway connects caregiver absence leading to care deficiency (S) with delayed assistance (T), reflecting process-related vulnerabilities in bedside care. A medication–behavior pathway links nighttime use of sedative-hypnotic drugs with toileting (N) to unassisted bed exit (U), representing a typical high-risk nighttime scenario. Additional links capture relationships between analgesia-related lower limb weakness (K → P), medication adherence and antiepileptic drug use (H → Q), and nursing management factors (A → B). Overall, the network structure illustrates recurrent patterns of conditional dependency among contributing factors documented in bed falls, rather than indicating deterministic causal mechanisms.

The learned network structure formed a directed acyclic graph (DAG) in which 13 variables were connected by directed edges, forming 10 directed relationships. The remaining nine variables did not exhibit statistically supported dependencies with other variables under the BIC scoring criterion and therefore appeared as isolated nodes in the learned network. Details of the network connectivity are shown in Figure 1 and Table 1.

**Table 1:** Node connectivity of contributing factors in the learned Bayesian network.

Variable	In-degree	Out-degree	Role in network
A	1	1	Intermediate node
B	1	0	Child node
C	0	1	Parent node
D	0	0	Isolated node
E	0	0	Isolated node
F	0	4	Parent node
G	2	0	Child node
H	1	1	Intermediate node
I	0	0	Isolated node
J	0	0	Isolated node
K	0	1	Parent node
L	0	0	Isolated node
M	0	0	Isolated node
N	0	1	Parent node
O	0	0	Isolated node
P	1	0	Child node
Q	1	0	Child node
R	0	0	Isolated node
S	1	1	Intermediate node
T	1	0	Child node
U	1	0	Child node
V	0	0	Isolated node

A, improper use of bed rails; B, inadequate postoperative nursing education; C, older adult patients using the toilet alone; D, improper use of call bells; E, patient’s auditory and visual impairments; F, sudden change in patient’s consciousness; G, orthostatic hypotension induced by diuretic antihypertensive drugs; H, poor drug adherence; I, inadequate monitoring of sedated patients; J, caregivers failing to notice patient’s condition in time; K, combined use of sedatives and femoral nerve block analgesia pumps; L, unsuitable bed design; M, poor collaboration between healthcare providers; N, using sedative-hypnotic drugs and using the toilet at night; O, patient’s cognitive and communication impairments; P, lower limb weakness caused by femoral nerve block analgesia pumps; Q, irregular use of antiepileptic drugs; R, impaired consciousness not being effectively managed; S, caregiver absence leading to care deficiency; T, caregivers not assisting patients in time; U, getting out of bed without assistance after using sedative-hypnotic and analgesic drugs; and V, non-slip resistant slippers.

This table summarizes the connectivity of all contributing factors in the learned Bayesian network. The in-degree and out-degree indicate the number of incoming and outgoing directed edges for each variable, respectively. Variables with both in-degree and out-degree equal to zero are classified as isolated nodes, indicating that no statistically supported dependencies with other variables were identified during the structure learning process.

#### 3.1.2 Bootstrap-based structural stability analysis

To assess the robustness of the learned network structure, bootstrap resampling was conducted on the original dataset. Bayesian network structure learning was repeated independently for each bootstrap sample using the same model configuration. The frequency with which each directed edge appeared across bootstrap samples was calculated to evaluate structural stability.

**Figure 2:**
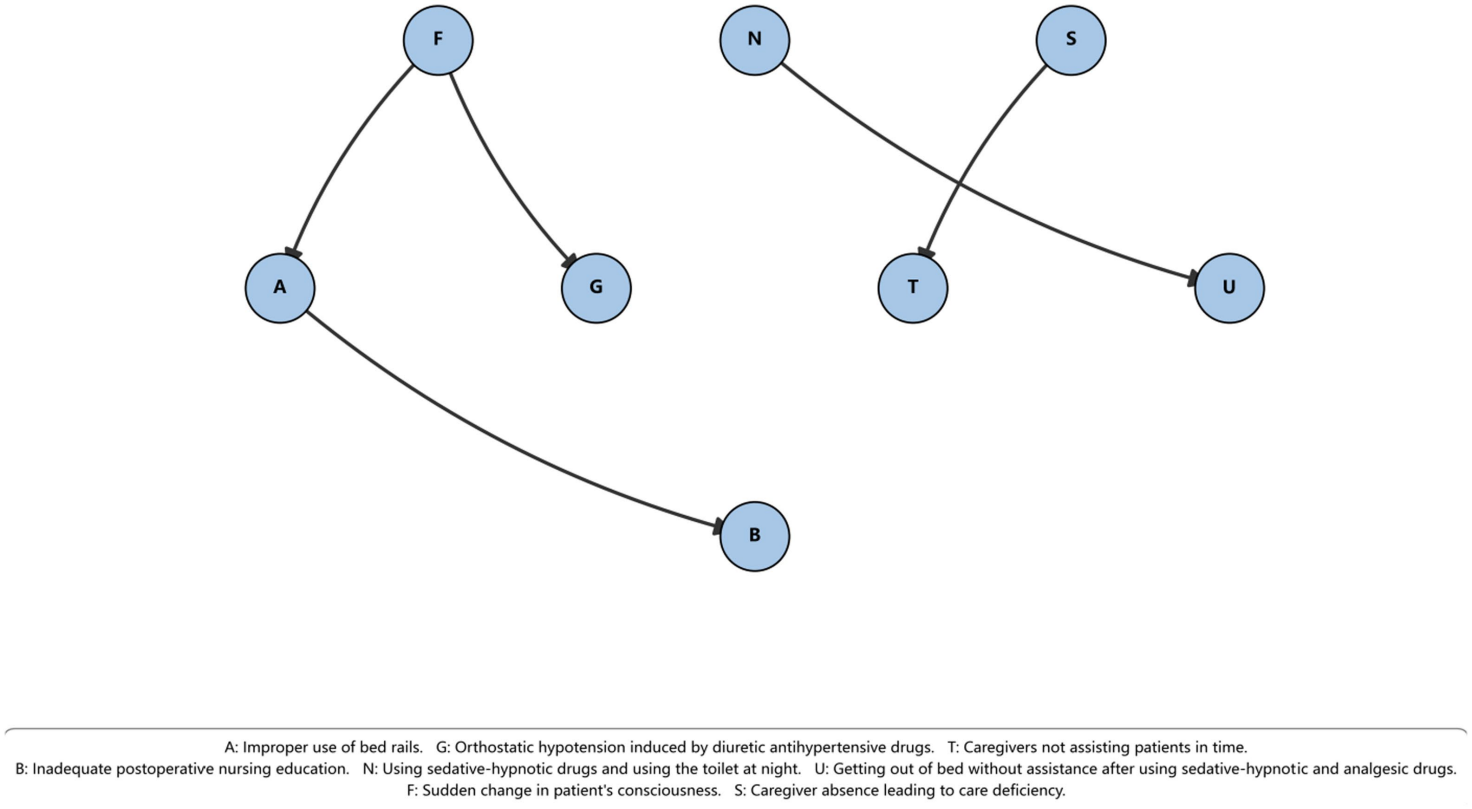
Stable Bayesian network structure of bed falls contributing factors identified through bootstrap analysis. This figure presents the stable Bayesian network obtained after bootstrap-based structural stability analysis. Five directed edges were retained as robust conditional dependency relationships: F → G, F → A, A → B, S → T, and N → U. Sudden change in patient consciousness (F) is conditionally associated with medication–related hypotension (G) and improper use of bed rails (A), with A further linked to inadequate postoperative nursing education (B). In addition, a caregiving pathway connects caregiver absence leading to care deficiency (S) with delayed assistance (T), while a medication–behavior pathway links nighttime sedative-hypnotic use with toileting (N) to unassisted bed exit (U).

As shown in Table 2 and Figure 2, the directed edge F → G exhibited the highest occurrence frequency (0.815), followed by S → T (0.745) and F → A (0.610). In addition, the edges A → B and N → U showed occurrence frequencies of 0.520 and 0.510, respectively. All other directed edges appeared in less than 10% of bootstrap samples. Based on these results, the five edges with relatively high occurrence frequencies (F → G, F → A, A → B, S → T, and N → U) were retained to construct the stable Bayesian network, which served as the basis for the subsequent conditional inference analysis.

**Table 2:** Directed edges with higher occurrence frequencies in bootstrap stability analysis.

From	To	Frequency	Count
F	G	0.815	163
S	T	0.745	149
F	A	0.610	122
A	B	0.520	104
N	U	0.510	102

F = sudden change in patient’s consciousness; G = orthostatic hypotension induced by diuretic antihypertensive drugs; S = caregiver absence leading to care deficiency; T = caregivers not assisting patients in time; A = improper use of bed rails; B = inadequate postoperative nursing education; N = using sedative-hypnotic drugs and using the toilet at night; U = getting out of bed without assistance after using sedative-hypnotic and analgesic drugs. Frequency indicates the proportion of bootstrap samples in which the directed edge was identified; Count indicates the absolute number of occurrences across 200 bootstrap iterations.

As summarized in Table 2, the most frequently identified directed edge across bootstrap resampling was F → G, followed by S → T and F → A. These edges consistently appeared in more than half of the bootstrap samples, indicating relatively stable conditional dependency relationships among the corresponding contributing factors.

In contrast, edges such as A → B and N → U demonstrated moderate occurrence frequencies, suggesting locally stable structural relationships within the network. All remaining directed edges appeared in fewer than 10% of bootstrap samples and were therefore not considered structurally stable for subsequent inference analyses.

#### 3.1.3 Results of conditional inference analysis

As shown in Table 3, conditional inference analyses quantified how the probabilities of selected contributing factors changed under different observed conditions.

**Table 3:** Summary of conditional inference result.

Condition	Condition counts (n/SUM)	Target	Target counts (n/SUM)	Baseline P(1) [95%CI]	Conditional P(1) [95%CI]	Absolute change	Relative change
F = 0	83/102	G	5/102	0.053 [0.024–0.102]	0.003 [0.003–0.003]	−0.050	−94.4%
F = 0	83/102	A	39/102	0.383 [0.277–0.471]	0.458 [0.355–0.563]	+0.075	+19.4%
F = 0	83/102	B	15/102	0.150 [0.083–0.219]	0.136 [0.077–0.203]	−0.014	−9.6%
A = 0	63/102	B	15/102	0.150 [0.083–0.219]	0.224 [0.129–0.335]	+0.074	+49.1%
S = 0	71/102	T	26/102	0.257 [0.180–0.335]	0.367 [0.258–0.478]	+0.110	+42.7%
N = 0	98/102	U	11/102	0.112 [0.053–0.180]	0.074 [0.024–0.135]	−0.038	−34.1%

F = sudden change in patient’s consciousness; G = orthostatic hypotension induced by diuretic antihypertensive drugs; A = improper use of bed rails; B = inadequate postoperative nursing education; S = caregiver absence leading to care deficiency; T = caregivers not assisting patients in time; N = using sedative-hypnotic drugs and using the toilet at night; U = getting out of bed without assistance after using sedative-hypnotic and analgesic drugs. Baseline P(1) represents the marginal probability of the target node being 1 without any condition. Conditional P(1) represents the probability of the target node being 1 given the specified condition. Absolute Change is calculated as Conditional P(1) minus Baseline P(1). Relative Change is calculated as the Absolute Change divided by Baseline P(1).

As shown in Table 2, fixing specific nodes to predefined states was associated with observable changes in the estimated probabilities of several related contributing factors within the network. When F was fixed at 0, the probability of G decreased markedly, while the probability of A increased and that of B showed a slight decrease, reflecting different probability changes within the F → A → B configuration. When A was fixed at 0, the probability of B increased substantially, suggesting a conditional association between these two variables. In addition, fixing S = 0 was associated with an increase in the probability of T, whereas fixing N = 0 was associated with a decrease in the probability of U. These results illustrate how the probabilities of different variables vary under specific observed conditions within the network.

For the relationship between sudden change in patient consciousness (F) and orthostatic hypotension induced by diuretic antihypertensive drugs (G), the observed frequency of G was 5/102 cases, indicating a low-frequency event in the dataset. Based on the Bayesian network model, the baseline probability of G was estimated as 0.053 (95% CI: 0.024–0.102). When F was fixed at 0, the conditional probability of G decreased to 0.003 (95% CI: 0.003–0.003), corresponding to an absolute decrease of 0.050 and a relative decrease of 94.4%.

For improper use of bed rails (A), the observed frequency was 39/102 cases. The baseline probability estimated by the model was 0.383 (95% CI: 0.277–0.471). Under the condition F = 0, the probability increased to 0.458 (95% CI: 0.355–0.563), corresponding to an absolute increase of 0.075. When improper use of bed rails was absent (A = 0), the probability of inadequate postoperative nursing education increased to 0.224 (95% CI: 0.129–0.335), compared with the baseline probability of 0.150.

Within the caregiving pathway, the observed frequency of delayed assistance (T) was 26/102 cases (25.5%). The baseline probability estimated by the model was 0.257 (95% CI: 0.180–0.335). When caregiver absence was not observed (S = 0), the probability increased to 0.367 (95% CI: 0.258–0.478).

Within the medication–behavior pathway, the observed frequency of unassisted bed exit (U) was 11/102 cases (10.8%), representing a relatively low-frequency event. The baseline probability estimated by the model was 0.112 (95% CI: 0.053–0.180). When nighttime sedative use with toileting did not occur (N = 0), the probability decreased to 0.074 (95% CI: 0.024–0.135).

These results illustrate how the probabilities of contributing factors vary under different observed conditions within the learned Bayesian network structure.

### 3.2 Results of posterior probability analysis

When G = 1 (orthostatic hypotension induced by diuretic antihypertensive drugs) was observed, the posterior probability of F = 1 (sudden change in patient’s consciousness) reached 0.955, indicating a strong co-occurrence pattern between consciousness alteration and hypotension-related events.

When U = 1 (getting out of bed without assistance after using sedative-hypnotic and analgesic drugs) was observed, the posterior probability of N = 1 (using sedative-hypnotic drugs and using the toilet at night) was 0.370, suggesting frequent co-occurrence of nighttime sedative use and toileting behavior in such events.

When T = 1 (caregivers not assisting patients in time) was observed, the posterior probability of S = 1 (caregiver absence leading to care deficiency) was only 0.009, indicating that delayed assistance more commonly occurred in situations where caregivers were present but unable to assist in time, rather than being entirely absent.

When B = 1 (inadequate postoperative nursing education) was observed, the posterior probability of F = 1 was 0.267, which was higher than that of A = 1 (0.081). This suggests that, in reports where inadequate postoperative nursing education was identified, patient-condition-related factors were more likely to co-occur than issues related to specific protective measure use.

## 4 Discussion

This study analyzed inpatient bed fall incident reports and used Bayesian network modeling to examine patterns of conditional dependencies among contributing factors within reported fall events. Bootstrap stability analysis identified several structurally consistent dependency configurations. Conditional inference and posterior probability analyses were then used to explore how the probabilities of related variables changed under different observed evidence conditions. Because the dataset consisted exclusively of reported bed falls without a non-fall comparison group, the present analysis does not identify epidemiologic contributing factors for falling in the hospitalized population. Instead, the model characterizes configurations of contributing factors observed within fall incidents themselves. The findings should therefore be interpreted as exploratory patterns within reported events rather than estimates of population-level fall risk.

First, sudden change in patient’s consciousness (F) occupied a central position in the network and exhibited stable directed connections to multiple other contributing factors within the reported incidents. Among these, the edge F → G (sudden change in patient’s consciousness → orthostatic hypotension induced by diuretic antihypertensive drugs) showed the highest occurrence frequency in the bootstrap analysis (0.815), indicating a highly stable conditional co-occurrence relationship between altered consciousness and medication-related orthostatic hypotension. It should be noted that the direction of this edge reflects the optimal data-driven structure under the BIC scoring criterion and does not necessarily imply a definitive temporal or causal ordering between altered consciousness and hypotension. Rather, this edge indicates a strong conditional co-occurrence pattern between these two clinically related states within the reported fall cases. The conditional inference results further showed that when F was fixed at 0, the occurrence probability of G decreased markedly (−94.4%), providing additional probabilistic support for the clinical plausibility of the above structural relationship. This finding is consistent with previous evidence that older adults using diuretics are prone to orthostatic hypotension due to reduced blood volume, which may lead to dizziness, transient impaired consciousness, and even falls; the fall risk among diuretic users has been reported to be approximately 1.18 times that of non-users (18). However, the existing literature also suggests that the direct association between antihypertensive medications and falls is not fully consistent: gait and balance impairment remains one of the strongest predictors of falls in older adults, and the use of multiple antihypertensive agents may increase fall risk only under certain conditions, with conclusions varying across studies (19). Taken together, our results suggest that the occurrence of bed falls is not determined by medication exposure alone, but may cluster under conditions where medication effects, physiological vulnerability, and altered consciousness co-occur.

However, orthostatic hypotension induced by diuretic antihypertensive drugs (G) was observed in 5 of the 102 reported bed falls (baseline probability = 0.053, 95% CI: 0.024–0.102). Given the relatively small number of observed G events, the estimated probabilities should be interpreted with caution. Although the relative change appeared large (−94.4%), the absolute probability difference remained modest because the baseline occurrence of G was low. In addition, the structural relationship between F and G showed relatively high stability in the bootstrap analysis, with the edge F → G identified in 81.5% of the resampled networks. This relatively high occurrence frequency suggests that the conditional dependency between these two variables represents a stable structural pattern within the analyzed incident dataset.

Second, among nursing management and safety education–related factors, the directed edge A → B (improper use of bed rails → inadequate postoperative nursing education) showed moderate structural stability in the bootstrap analysis (frequency = 0.520). Conditional inference indicated that when improper bed rail use was not reported (A = 0), the probability of inadequate postoperative nursing education (B) increased substantially (+49.1%). Consistently, when sudden changes in patient consciousness did not occur (F = 0), the probability of improper use of bed rails (A) increased (+19.4%), while inadequate postoperative nursing education (B) slightly decreased (−9.6%). This pattern suggests that bed falls in postoperative patients may arise from different underlying situations. In some cases, falls are associated with improper bed-rail use as a physical safety issue, whereas in others they appear more closely related to insufficient postoperative instructions about when to request assistance during early mobilization. In routine care, nurses may need to regularly check whether bed rails are appropriately positioned and clearly remind postoperative patients to request assistance before attempting to get out of bed during early mobilization (20–22).

Within the caregiving configuration of contributing factors observed in the reported fall incidents, the directed link from caregiver absence leading to care deficiency (S) to caregivers not assisting patients in time (T) showed high structural stability in the bootstrap analysis (occurrence frequency = 0.745). Conditional inference indicated that when S = 0, the T increased substantially (+42.7%). This association suggests that fall events occurring at the bedside may also be related to care workflow organization and response timeliness, rather than being determined solely by whether caregivers are physically present. Previous studies have shown that even when nursing staff or family caregivers are present in the ward, high workload, multitasking, or attentional distraction may impair timely recognition of patients’ immediate risk signals, resulting in delays in necessary protective or assistive actions (23). From a practical perspective, this finding suggests that delayed assistance may occur even when caregivers are physically present in the ward, particularly during periods of high workload or task concentration. In routine nursing practice, such situations often arise during shift handovers, medication preparation, or other time-intensive care activities when staff attention is temporarily focused on specific tasks. One practical approach may therefore be to anticipate these busy periods and conduct brief patient rounds beforehand to check whether patients need assistance with activities such as toileting or bed exit. Addressing potential needs in advance may help reduce situations in which patients attempt to leave the bed without assistance while staff are occupied with other duties.

Within the medication-behavior pathway, the directed relationship N → U showed relatively stable structural features in the bootstrap analysis (occurrence frequency = 0.510), indicating that patients who use sedative-hypnotic medications at night and go to the toilet independently are more likely to attempt getting out of bed without assistance. This finding suggests that bedside fall risk is not driven solely by medication effects or physiological changes, but is substantially amplified through the interaction between patient behavior and medication-related functional impairment. Previous studies have reported that various central nervous system medications-including anxiolytics, hypnotics, antipsychotics, opioid analgesics, and antiepileptic drugs are associated with an increased risk of falls. Among these, traditional benzodiazepine receptor agonists have been identified as important and modifiable contributing factors for falls in hospitalized older adults, whereas some newer hypnotic agents may offer a relatively safer profile (24, 25). In addition, nocturia itself is a well-established predictor of falls in older adults, with approximately 80% of nocturnal toileting-related falls occurring in situations where patients attempt to get out of bed without assistance, most frequently during the high-risk time window between 23:00 and 01:00 (26, 27). Accordingly, when sedative or hypnotic medications impair cognitive or motor function and coincide with nighttime toileting behavior, the risk of unassisted bed exit is further increased. These findings highlight the need for targeted nighttime nursing strategies for patients with such medication and behavioral characteristics, including enhanced night-time surveillance, reinforcement of calling for assistance before ambulation, provision of bedside commodes or toilet chairs, assessment of assistance needs prior to medication administration, and routine evaluation of nocturia risk at admission (27).

## 5 Limitation

This study employed a data-driven Bayesian network model without imposing prior structural constraints. The learned network structure was therefore used for exploratory analysis and hypothesis generation rather than for strict causal inference. In addition, this study was based on retrospective fall incident reports from a single center with a relatively limited sample size. Consequently, the generalizability and robustness of the findings should be further validated in larger, multi-center studies.

Another limitation relates to the nature of the incident-report documentation. The contributing factors analyzed in this study were coded from narrative descriptions recorded in routine incident reports. Because these reports are completed by different staff members and often under varying clinical circumstances, the level of detail and the way events are described may differ across reports. Such variation in documentation could influence how factors were identified and coded as binary variables in the dataset. In turn, this may influence the dependency structure inferred by the Bayesian network model. Nevertheless, the analysis remains useful for exploring relationships among contributing factors observed in reported bed falls, and the findings may help guide further research using larger and more standardized datasets.

Finally, the network structure was learned from 102 observations across 22 binary variables. This represents a relatively sparse data setting for structural learning, which may affect the stability of the learned network. Under these conditions, alternative network structures might also provide similar fits to the data. Therefore, the identified dependency patterns should be interpreted as exploratory and require confirmation in larger datasets.

## 6 Implication

The findings of this study suggest that sudden changes in patients’ consciousness represent a common structurally central node across multiple structurally consistent contributing-factor configurations. In clinical practice, this implies that transient confusion, drowsiness, or delayed responsiveness should be treated as a critical “risk signal,” rather than as an isolated symptom. When such changes are observed, attention should extend beyond routine neurological or vital-sign monitoring to include a timely reassessment of medication use, mobility status, and the appropriateness of existing protective measures.

Analysis of the medication-behavior pathway further indicates that the combination of nighttime sedative use and toileting needs constitutes a particularly a common contributing-factor context observed in reported bedside fall incidents. This finding has clear practical implications: for patients receiving sedative-hypnotic medications who also have nocturia, fall prevention strategies should incorporate targeted behavioral support measures. These include explicitly reinforcing the need to call for assistance before getting out of bed at night, proactively providing bedside commodes or urinals, and increasing observation during high-risk nighttime periods, rather than relying solely on routine verbal education or generic fall-prevention instructions.

In addition, results from the caregiving process pathway indicate that delayed assistance is not simply attributable to caregiver absence, but more often occurs when caregivers are present but unable to respond promptly. This observation has important implications for nursing management. Bedside fall prevention is not only a matter of staff presence, but is closely related to staffing patterns, workload, and response mechanisms. Accordingly, nursing managers may consider optimizing personnel allocation during high-risk periods and clarifying response workflows, in order to reduce delays caused by competing tasks or excessive workload.

## 7 Conclusion

This study used Bayesian network modeling to examine the conditional dependency structure among contributing factors associated with reported bed falls in hospitalized patients. By combining bootstrap stability assessment with conditional and posterior probability analyses, several structurally consistent contributing-factor configurations were identified. The findings indicate that bed falls rarely result from single factors, but rather occur when a small number of key contributing factors co-occur under specific clinical conditions.

Sudden changes in patients’ level of consciousness appeared as a central node in the network, showing connections with multiple factors and suggesting a strong association with reported bed falls. In addition, the combination of night-time sedative-hypnotic use and toileting behavior was associated with a higher probability of unassisted bed exit, representing a typical structurally consistent contributing-factor configurations during night-time care. In addition, delayed assistance was more likely to occur when caregivers were present but unable to respond promptly, highlighting the importance of response efficiency in addition to caregiver availability.

Overall, these results provide a structural and scenario-based perspective on bed-fall risk, offering clinically relevant insights for targeted risk assessment and prevention.

## Data Availability

The raw data supporting the conclusions of this article will be made available by the authors without undue reservation.
